# Supplementary material for: The Relationship between Numbness and Quality of Life
Source: J Clin Med. 2023 Feb 7;12(4):1324. doi: 10.3390/jcm12041324 (PMC9965061; doi:10.3390/jcm12041324)
Supplement: Supplementary file 1 [file jcm-12-01324-s001.zip › jcm-2152938-supplementary.pdf]

2013-09028

To the right is the number we need to send you a thank you. Please do not cut it off.

2013 Questionnaire about your body

The Japanese Orthopaedic Association

October 2013

The Japan Research Center is a specialized organization for surveys and research that collects your opinions, compiles the results into various statistics, and serves as a bridge to public organizations and companies.

The Japan Research Center has been commissioned by the **Japanese Orthopaedic Association** to conduct a "Questionnaire on Physical Pain. This survey is part of an academic research project, asking for the opinions of the general public about the pain they experience on a daily basis.

All of your responses will be summarized in a statistical number, such as "______percent of the responses".

Your name will never be mentioned.

We will not use your opinion for sales or any other purpose.

Japan Research Center, Inc. is a research company that conducts research on the following topics in accordance with the Act on the Protection of Personal Information and the Japan Marketing Research Association.

We conduct public opinion and market research in compliance with the Marketing Research Code of Conduct.

We sincerely appreciate your understanding of the purpose of this survey and sincerely appreciate your cooperation.

Those who complete the survey will receive a library card worth 500 yen by the end of November.

The Japanese Orthopaedic Association

Research Institute Japan Research Center, Inc.

Japan Research Center Inc.
Gallup International Member
Res[illegible]arge: Atsushi Hagiwara and
Atsushi Suzuki
Toll free 0120-030-551 (Weekdays 10:00-17:00)
(Closed at 12:00-13:00)
2-7-1 Nihonbashi-Honcho, Chuo-ku, Tokyo 103-0023, Japan
Home Page http://www.nrc.co.jp

JMA QMS REGISTRATION CENTER
JMAQA-2418
たいせつにしますプライバシー
12390009(07)

<To answer the questionnaire, please read the following.

- The person to whom this questionnaire is addressed is the one who should complete it.
- Please circle the questions in the order of their number in pencil or with a black or blue ballpoint pen according to the instructions.
- Please circle the number(s) that apply to you. Please circle only one answer per question. Please respond according to the instruction "circle as many as you like."
- **Please be sure to answer all of the questions in the "One of each" and "Any number of each" sections, as these questions are for each of these items.**
- For some questions, we may ask you to provide the answer number itself or a specific number.
- If "Other (Other)" applies, please be as detailed as possible. If "Other" applies, please provide as much detail as possible in parentheses (Please provide as much detail as possible in parentheses ( ).

Please return the completed survey form in the enclosed return envelope to

We will ask you about your body's "pain

[To all].

the ninth month of the lunar calendar

Q 1 Have you ever had neck pain, stiff shoulders, back pain, limb pain, or any other pain caused by bones, muscles, joints, or nerves?

Have you ever experienced pain that you think you might have had? (Exclude menstrual pain and toothache) (circle only one)

**1** Yes	**2** No.

→ Please go to page 8 [QUESTION B

↓ Please proceed to [QUESTION A] below

[Question A]

For the following questions, please answer the **"pain you have experienced" that you answered in Q1.**
If your pain is caused by more than one disease, please answer about the pain that is

Q 2 When was the most recent time you experienced the pain? (circle only one)

(If you have more than one location, please select the one you are having the most trouble with.)

1 today
2 Not today, but within a week
3 More than 1 week, less than 1 month
4 Within 1–3 months
5 Within 4–6 months
6 More than 6 months ago

Q 3 How often does the pain hurt? Which of the following best describes your pain? (circle only one)

(If you have more than one location, please select the one you are having the most trouble with.)

1 Always in pain.
2 1 Pain during the day, but not always
3 Several times a day
4 About once a day
5 2–3 times a week
6 About once a week
7 About once a month
8 Less than once a month

Q 4 How long has the pain lasted?

(If you have more than one location, please select the one you are having the most trouble with.)

[ ][ ] About a year and a month [ ][ ] (Fill in the numbers)

Q5- 1 Where is the location of the pain? Please paint all of the pain areas black. (as many as you can)

(Please refer to the table on the right for the name of the site.)

*Reference

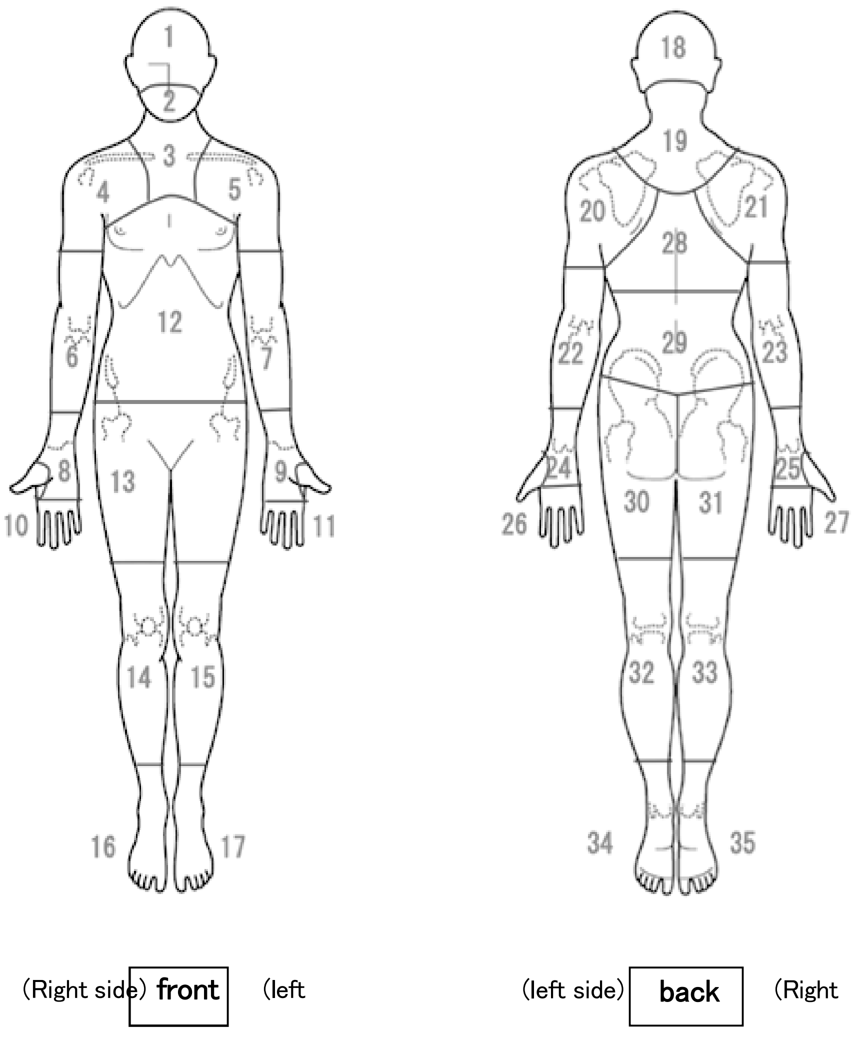

[Front of the body].

1 head
2 jaw
3 neck
4 right shoulder
5 left shoulder
6 Right elbow and surrounding area
7 Left elbow and surrounding area
8 Right wrist and right palm
9 Left wrist and left palm
10 right hand
11 left hand
12 Chest, abdomen
13 Hips and thighs
14 Right knee and surrounding area
15 Left knee and surrounding area
16 Right ankle, instep, and toe
17 Left ankle, instep, and toe

Back of the body]

18 Head (back of head)
19 Neck (posterior neck)
20 left shoulder
21 right shoulder
22 Left elbow and surrounding area
23 Right elbow and surrounding area
24 Left wrist and back of left hand
25 Right wrist and back of right hand
26 left hand
27 right hand
28 back
29 back
30 Left buttock and left thigh
31 Right buttock and right thigh
32 Back of left knee and calf area
33 Back of right knee and calf area
34 Left ankle, heel, sole and toe
35 Right ankle, heel, sole and toe

Q5- 2

Paint **one (1) of the most painful** areas in black. (Only one)

If the most painful area **spans more than one site**, select and apply to **only one of the most painful areas.**

(Please refer to the table on the right for the name of the site.)

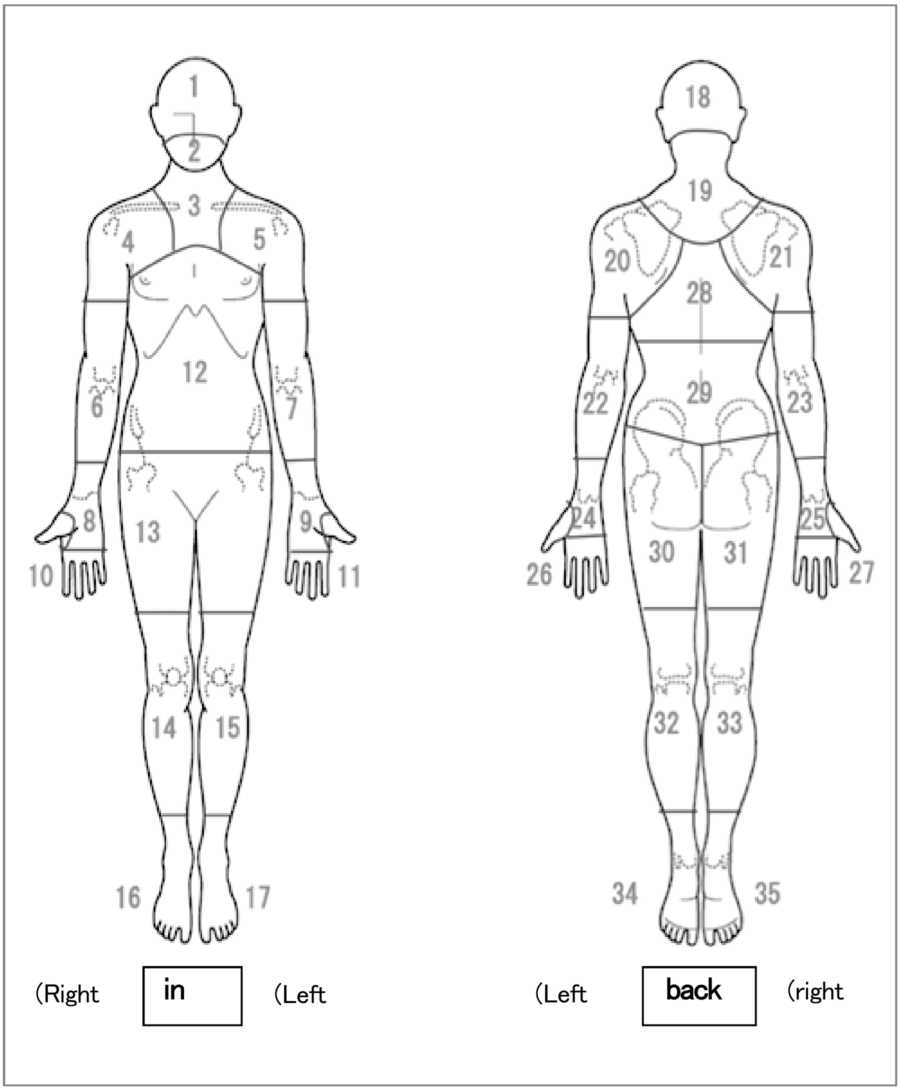

*Reference

[Front of the body].

1 head
2 jaw
3 neck
4 right shoulder
5 left shoulder
6 Right elbow and surrounding area
7 Left elbow and surrounding area
8 Right wrist and right palm
9 Left wrist and left palm
10 right hand
11 left hand
12 Chest, abdomen
13 Hips and thighs
14 Right knee and surrounding area
15 Left knee and surrounding area
16 Right ankle, instep, and toe
17 Left ankle, instep, and toe

Back of the body]

18 Head (back of head)
19 Neck (posterior neck)
20 left shoulder
21 right shoulder
22 Left elbow and surrounding area
23 Right elbow and surrounding area
24 Left wrist and back of left hand
25 Right wrist and back of right hand
26 left hand
27 right hand
28 back
29 back
30 Left buttock and left thigh
31 Right buttock and right thigh
32 Back of left knee and calf area

Q 6 Please tell us the intensity of the pain in the 'most painful place' that you answered in Q5-2.

On a scale of "0" for no pain and "10" for the worst pain imaginable, how bad is your pain?

Please circle the appropriate number below. (circle only one)

0	1	2	3	4	5	6	7	8	9	10

No pain ←——————————————————→ I can imagine. WorstPain

Q 7 For the 'most painful place' you answered in Q5-2, please select one disease name from the following that causes the pain.

(Only one circle)

[Pain due to nerve damage] [Pain in other muscles, bones, and joints

spinal fluid solution
1 Pain after spinal cord injury
UWBWS
2 Lumbar spinal canal stenosis
lumbosacral lumbar region
3 Lumbar disc herniation
cervical vertebrae root
4 **C e r v i c a l** Neuropathy
unmanageable person (esp. a woman)
5 Herniated **c e r v i c a l** disc
bruise
6 Sciatica
difference
7 Trigeminal neuralgia
herpes zoster herpes zoster
8 Postherpetic neuralgia, herpes zoster
six ministries (of Zhou-dynasty China)
9 Intercostal neuralgia
minor details
10 Peripheral neuritis
the Lord's Prayer Room (a.k.a. "The Lord's Prayer Room") central bureaucracy
11 Carpal Tunnel Syndrome and Elbow Tunnel Syndrome
12 Multiple sclerosis
an informal relationship between a child and his or her parents
13 Spinal Cord Syringomyelia
14 Diabetic neuropathy
15 Alcoholic neuropathy
transpiration
16 Post-stroke pain
monopoly
17 Phantom limb pain and disconnection pain
18 Reflex sympathetic dystrophy (CRPS type1)
19 Cauzalgy (CRPS type 2)
main purpose permeation
20 Neuropathy due to nerve compression or invasion by tumor
minor details
21 Drug-induced peripheral neuropathy, vitamin deficiency, and amyloidosis
22 Post-irradiation plexopathy
nerve tissue
23 Pain after brachial plexus withdrawal injury
24 Surgical nerve damage (spinal surgery)
25 Surgical nerve damage (non-spinal surgery)

26 lumbago
27 stiff shoulders
28 osteoarthritis of the knee
29 osteoarthritis of the hip
30 frozen shoulder, periarthritis of shoulder joint bursitis
bursitis
31 tendinitis
tendonitis
32
33 Peripheral vascular disease rheumatoid arthritis
peripheral peripheral peripheral peripheral peripheral
34 (peripheral circulatory failure)
tendonitis
35 Tennis elbow and tendonitis
36 arthritis
sprained joint
(Japanese) stone coffin
37 Cancer/Malignancies
seed leaf
38
39 infection Cervical sprain
Cuneiform (constellation)
40 (whiplash) Pain from
spine
41 spinal surgery
42 Pain after artificial joint surgery
fibrofibrillary artery syndrome
43 fibromyalgia
44 Other (other))
45 I don't know the name of the disease.
